# Mutant p53 dictates the oncogenic activity of c-Abl in triple-negative breast cancers

**DOI:** 10.1038/cddis.2017.294

**Published:** 2017-06-29

**Authors:** Chevaun D Morrison, Jenny C Chang, Ruth A Keri, William P Schiemann

**Affiliations:** 1 Case Comprehensive Cancer Center, Division of General Medical Sciences-Oncology, Case Western Reserve University, Cleveland, OH 44106, USA; 2 Houston Methodist Research Center, Houston, TX 77030, USA; 3 Department of Pharmacology, Case Western Reserve University, Cleveland, OH 44106, USA

## Abstract

We recently established c-Abl as a potent suppressor of triple-negative breast cancer (TNBC) progression through its reactivation of a p53:p21 signaling axis coupled to senescence. Moreover, we observed co-expression of p53 and c-Abl to be essential for normal mammary epithelial cell physiology, as this relationship is lost upon breast cancer progression. Cytoplasmic c-Abl activity is markedly increased in some TNBCs and contributes to disease progression; however, the mechanisms underlying these events remain largely unknown. In addressing this question, we show here that c-Abl is predominantly restricted to the cytoplasm of human MDA-MB-231 TNBC cells, and to the nucleus of human MCF-7 luminal A cells. TTK is a mitotic protein kinase that phosphorylates c-Abl on Thr735, thereby creating a recognition binding motif for 14-3-3 adaptor proteins in response to oxidative stress. By interrogating the METABRIC database, we observed a significant correlation between p53 expression and that of c-Abl and TTK in basal-like breast cancers. Moreover, heterologous expression of TTK in MCF-7 cells significantly stimulated their growth in part via a c-Abl-dependent mechanism. Conversely, depleting TTK expression in MDA-MB-231 cells not only inhibited their organoid growth in 3D-cultures, but also sensitized them to the tumor suppressing activities of c-Abl independent of its subcellular localization. Moreover, we show that mutant p53 forms cytoplasmic complexes with c-Abl, thereby dictating the subcellular localization of c-Abl and the sensitivity of MDA-MB-231 cells to Imatinib. In response to nutrient deprivation, c-Abl:p53 complexes readily accumulate in the nucleus, resulting in the hyperactivation of c-Abl and initiation of its anti-tumor activities. Collectively, we identified a novel mutant p53:c-Abl cytoplasmic signaling complex that promotes MDA-MB-231 cell growth and highlights the contextual cues that confer oncogenic activity to c-Abl in breast cancer.

c-Abl is a ubiquitously expressed nonreceptor protein tyrosine kinase (PTK) that exhibits distinct cellular functions depending upon whether this PTK localizes to the cytoplasm or nucleus. For instance, cytoplasmic c-Abl predominantly stimulates cell survival in response to growth factors; however, during mammary gland development, epidermal growth factor (EGF) deprivation promotes Mig6:c-Abl dependent apoptosis to regulate normal mammary epithelial cell (MEC) physiology.^1^ Alternatively, DNA damage readily induces the accumulation of c-Abl in the nucleus where it either inhibits cell growth or promotes apoptosis depending on the extent of DNA damage.^2^ These disparate activities of c-Abl likely underlie its dichotomous roles in regulating the development and progression of solid tumors. For instance, as MECs acquire malignant phenotypes, the function of c-Abl to either suppress or promote mammary tumorigenesis becomes highly controversial. Thus, although a number of oncogenic functions have clearly been attributed to c-Abl in human breast cancers,^3, 4, 5, 6, 7, 8^ we and others have observed activation of c-Abl to serve as an essential suppressor of breast cancer progression,^9, 10, 11^ particularly their acquisition of EMT and metastatic phenotypes.

A growing body of work in the scientific literature has enumerated a variety of oncogenic activities for c-Abl; however, the specific context in which the tumor suppressing activities of c-Abl are hijacked to drive breast cancer progression is largely unknown. We previously demonstrated the ability of c-Abl to inhibit oncogenic TGF-*β* signaling in TNBCs,^10^ an event requiring c-Abl to activate an autocrine TGF-*β*1-dependent Smad2:Smad1/5/8 signaling axis that reactivated p53 expression and led to the induction of a p21-dependent senescent reaction.^11^ Interestingly, although c-Abl and p53 are co-expressed in normal mammary tissues, we observed both proteins to be expressed in a highly discordant manner in human breast cancers. Importantly, stratifying breast cancer patients by dual c-Abl and wild-type p53 expression trended to be a more powerful prognostic indicator of patient survival as compared to singular stratification of c-Abl.^11^ These findings suggest that p53 expression and its mutational status could be an important contributor to the functional output of c-Abl across different breast cancer subtypes and contexts. Unfortunately, the extent to which p53 status correlates with the tumor activities of c-Abl in breast cancer remains unknown. As such, we hypothesized that the ability of c-Abl to suppress TNBCs development and progression is dependent upon its co-expression with wild-type p53. A corollary states that breast cancers, which escape c-Abl-mediated suppression, do so through discordant expression of c-Abl and wild-type p53, or alternatively through mutational inactivation of p53 to disrupt its normal functions. Therefore, the objective of this study was to determine whether mutant p53 expression contributed either directly or indirectly to the loss of c-Abl mediated suppression of TNBC.

## Results

### Differential localization and activity of c-Abl in luminal *versus* triple-negative breast cancer cells

**Figure 1:**
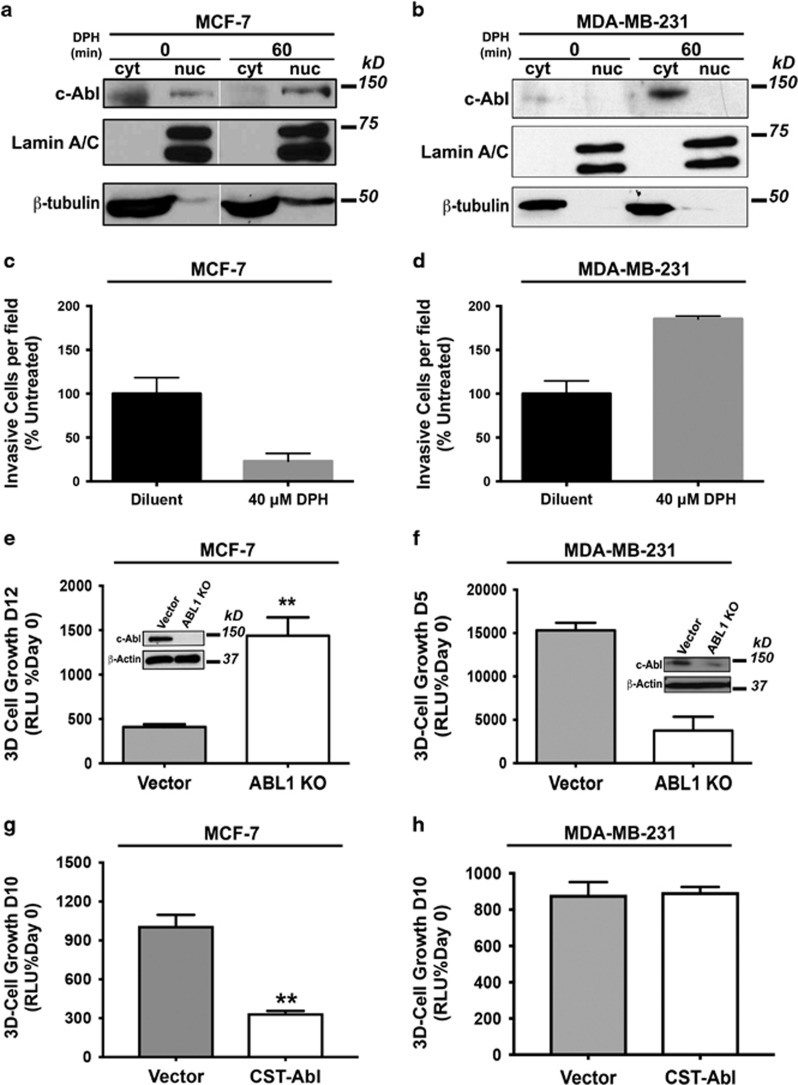
Differential localization and activity of c-Abl in luminal *versus* triple-negative breast cancer cells. (**a** and **b**) Administration of the small molecule c-Abl activator, DPH (20 *μ*M) induced the accumulation of c-Abl in the nucleus in MCF-7 cells (**a**), and the cytoplasmic stabilization of c-Abl in MDA-MB-231 cells (**b**). Data are representative of three independent analyses. (**c** and **d**) DPH (40 *μ*M)-mediated activation of c-Abl inhibited the invasion of MCF-7 cells (**c**) and stimulated that of MDA-MB-231 cells (**d**). Data are the mean (±S.D.) of two independent experiments completed in triplicate. (**e** and **f**) CRISPR/Cas9-mediated ABL1 knockout significantly induced MCF-7 organoid growth in 3D-cultures (**e**), whereas loss of ABL1 function markedly reduced MDA-MB-231 organoid growth (**f**). Data are the mean (±S.E.) of three independent experiments completed in triplicate for **e** and the mean (±S.D.) of two independent experiments completed in triplicate for **f**. (**g** and **h**) CST-Abl expression significantly attenuated MCF-7 organoid growth, but failed to impact that of MDA-MB-231 organoids (**h**). Data are the mean (±S.D.) of a representative experiment of three independent experiments completed in triplicate for **g** and **h**. **P<0.01

We recapitulated previous findings that showed c-Abl to possess divergent cellular localizations in MCF-7 (primarily nuclear) *versus* MDA-MB-231 (predominantly cytoplasmic) cells (Figures 1a and b). Along these lines, activating c-Abl by administration of the small molecule c-Abl activator, DPH,^12^ readily increased the accumulation of c-Abl in the nuclei of MCF-7 cells (Figure 1a), as well as inhibited their invasion through reconstituted basement membranes (Figure 1c). In stark contrast, treating MDA-MB-231 cells with DPH stabilized c-Abl expression in the cytoplasm (Figure 1b) and induced MDA-MB-231 cell invasion (Figure 1d). Similarly, CRISPR/Cas9-mediated knockout of *ABL1* in these same cell lines stimulated the proliferation of MCF-7 cells (Figure 1e) and inhibited that of MDA-MB-231 cells (Figure 1f). Finally, expressing a constitutively active mutant of c-Abl (CST-Abl) significantly attenuated MCF-7 organoid growth (Figure 1g), whereas MDA-MB-231 organoid growth failed to be impacted in response to CST-Abl expression (Figure 1h). Taken together, these findings highlight the differential activities elicited by c-Abl in luminal A (MCF-7) and TNBC (MDA-MB-231) cells, and in doing so, points to a role for nuclear c-Abl in mediating tumor suppression and cytoplasmic c-Abl in mediating tumor promotion.

### p53 expression dictates a significant correlation between c-Abl and the mitotic kinase TTK in breast cancer

**Figure 2:**
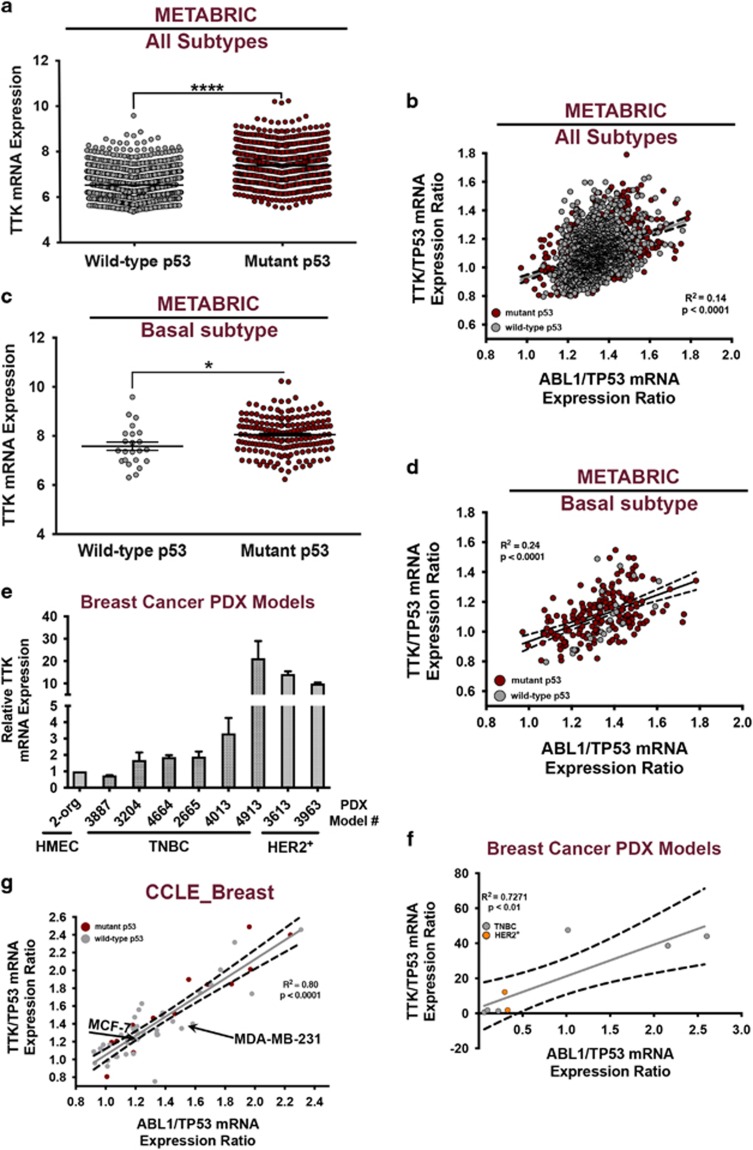
p53 expression dictates a significant correlation between c-Abl and the mitotic kinase TTK in breast cancer. (**a**) Analysis of the METABRIC breast cancer data set comparing TTK expression in wild-type and mutant p53-expressing tumors across all breast cancer subtypes. ****P<0.0001. (**b**) Linear regression analysis of the METABRIC breast cancer data set comparing the correlation between TTK:TP53 and ABL1:TP53 expression ratios. (**c**) Comparison of TTK expression in wild-type and mutant p53-expressing basal-like breast cancer patients from the METABRIC breast cancer data set. *P<0.05. (**d**) Linear regression analysis of the basal-like subtype of the METABRIC breast cancer data set comparing the correlation between TTK:TP53 and ABL1:TP53 expression ratios. (**e**) TTK expression in a cohort of TNBC and HER2^+^ breast cancer PDX models. (**f** and **g**) Linear regression analysis comparing the correlation between TTK:TP53 and ABL1:TP53 expression ratios in the PDX breast cancer models (**f**), and in breast cancer cell lines from the Cancer Cell Line Encyclopedia (CCLE; **g**). MCF-7 (ABL1:TP53 ratio=1.199; TTK:TP53 ratio=1.313) and MDA-MB-231 (ABL1:TP53 ratio=1.462 and TTK:TP53 ratio=1.514) are indicated in **g**

Previous findings observed c-Abl to reside predominantly in the cytoplasm of TNBC cell lines,^13^ which contrasts sharply with the nearly exclusive nuclear localization of c-Abl in normal human breast tissues.^11^ At present, the cellular and molecular events that govern this divergence in c-Abl localization remain to be fully elucidated. It should be noted that in response to oxidative stress, c-Abl is phosphorylated by the mitotic kinase TTK on Thr735, thereby creating a 14-3-3 docking site that may sequester c-Abl within the cytoplasm and prevent its nuclear targeting.^14^ Importantly, aberrant TTK expression has been observed in mutant p53-positive TNBCs, an event that is significantly correlated with elevated risk of relapse and docetaxel resistance.^15, 16, 17, 18^ In light of these findings, we hypothesized that aberrant TTK expression inhibits the tumor suppressive functions of c-Abl by facilitating its cytoplasmic retention in TNBCs that express mutant p53. Accordingly, we found TTK to be significantly overexpressed in breast cancers that express mutant p53 as compared to their wild-type expressing counterparts, an association that occurred independent of breast cancer subtype (Figure 2a; METABRIC data set^19^). This finding led us to suspect that p53 serves as a molecular determinant of TTK and c-Abl function in breast cancers. To address this supposition, we compared the correlation between expression ratios of TTK to p53, as well as of c-Abl to p53. In doing so and in accounting for the variability in p53 expression, we found TTK and c-Abl expression to have a direct and significant correlation across all breast cancer subtypes in the same patient cohort (Figure 2b). Consistent with the finding that (i) mutant p53 expression is a defining characteristic of basal-like BC, and (ii) aberrant TTK expression is directly correlated with that of mutant p53, we also observed TTK expression to be significantly elevated in mutant p53-expressing basal-like BC patients (Figure 2c; Pam50 filter of METABRIC data set). Likewise, the p53-dependent association of TTK and c-Abl expression was more tightly correlated in the basal-like BC subtype, highlighting its relevance specifically within this disease context (Figure 2d).

To expand the potential clinical relevance of these associations, we next monitored TTK expression in a cohort of TNBC and HER2^+^ PDX models^20^ and observed markedly upregulated TTK expression in both breast cancer subtypes (Figure 2e), an event that was also significantly correlated with c-Abl expression in a p53-dependent manner (Figure 2f). It is interesting to note that this association also held true for HER2^+^ breast cancers (Supplementary Figure S1a; METABRIC data set), whereas the expression of mutant p53 failed to correlate with that of TTK (Supplementary Figure S1b). These findings suggest that the ratio at which c-Abl and p53 are expressed may be a more relevant predictor of TTK overexpression; they also indicate that discordant expression profiles between c-Abl and p53 may participate in driving the progression of HER2+ breast cancers as well. Finally, we interrogated the Cancer Cell Line Encyclopedia (CCLE) database and observed a similarly significant p53-dependent correlation between TTK and c-Abl expression in breast cancer cell lines as compared to the aforementioned clinical data sets (Figure 2g). Included in these analyses were MCF-7 (luminal A) and MDA-MB-231 (basal-like) cell lines, which clearly express divergent levels of p53, TTK, and c-Abl (Figure 2g). Indeed, MCF-7 cells maintain higher levels of c-Abl and wild-type p53 and do not overexpress TTK, whereas MDA-MB-231 cells express lower levels of c-Abl and are mutant p53 positive, and thus overexpress TTK (Figure 2g). Collectively, these findings support a role for p53 in governing TTK and c-Abl expression, and in regulating c-Abl functions in genetically distinct breast cancer subtypes.^13^

### The tumor promoting activities of TTK manifest in part via a c-Abl-dependent mechanism

**Figure 3:**
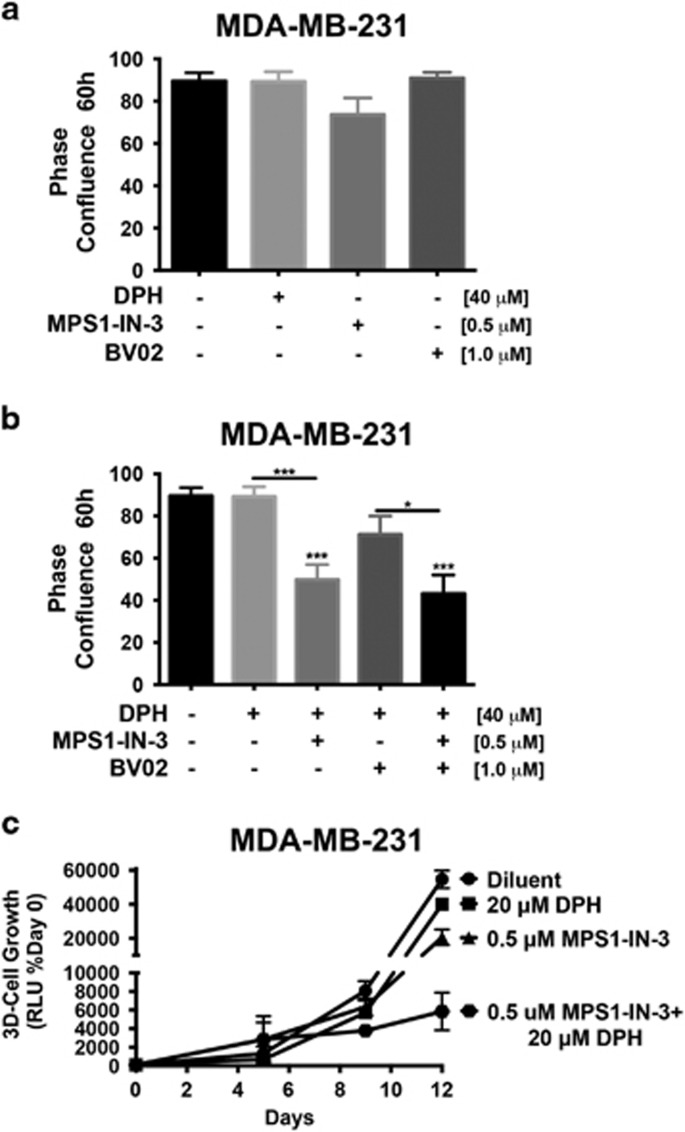
TTK inhibition sensitizes MDA-MB-231 cells to DPH-mediated c-Abl activation. (**a** and **b**) MDA-MB-231 cells were incubated for 60 h in the absence or presence of MPS1-IN-3 (0.5 *μ*M), BV02 (1 *μ*M) or DPH (40 *μ*M) either singly or combined as indicated. Differences in cell growth were analyzed on using the IncuCyte Zoom phase-contrast analysis package. Data are the mean (±S.E.) of three independent experiments completed in triplicate. *P<0.05; ***P<0.001. (**c**) MDA-MB-231 organoid growth in 3D-cultures was determined in the absence or presence of MPS1-IN-3 or DPH either singly or combined as indicated. Differences in longitudinal growth were quantified by firefly bioluminescence. Data are the mean (±S.D.) of one representative experiment that was performed twice in triplicate

**Figure 4:**
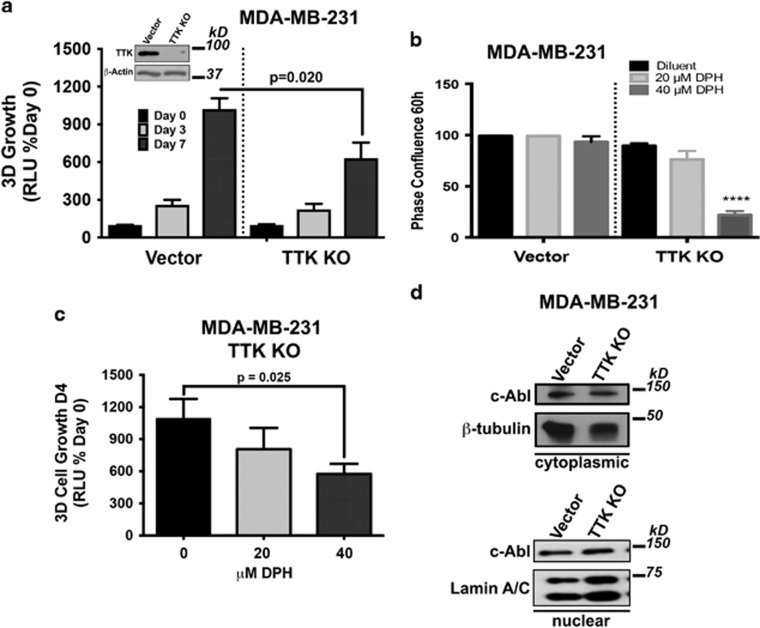
CRISPR/Cas9-mediated TTK knockout significantly inhibits MDA-MB-231 cell proliferation and sensitizes them to DPH-mediated c-Abl activation. (**a**) CRISPR/Cas9-mediated TTK knockout significantly inhibited MDA-MB-231 organoid growth in 3D-cultures. Inset confirms TTK knockout by immunoblot analyses. (**b** and **c**) Loss of TTK expression sensitizes MDA-MB-231 cells to the inhibitory effects of DPH (20 and 40 *μ*M) in 2D-cultures as assessed by IncuCyte Zoom phase-contrast analysis package (**b**), and in 3D-cultures by firefly bioluminescence (**c**). (**d**) Western blot analyzing nuclear c-Abl expression after the cellular fractionation of MDA-MB-231 CRISPR/Cas9 derivatives. Data are the mean (±S.E.) of three independent experiments completed in triplicate

**Figure 5:**
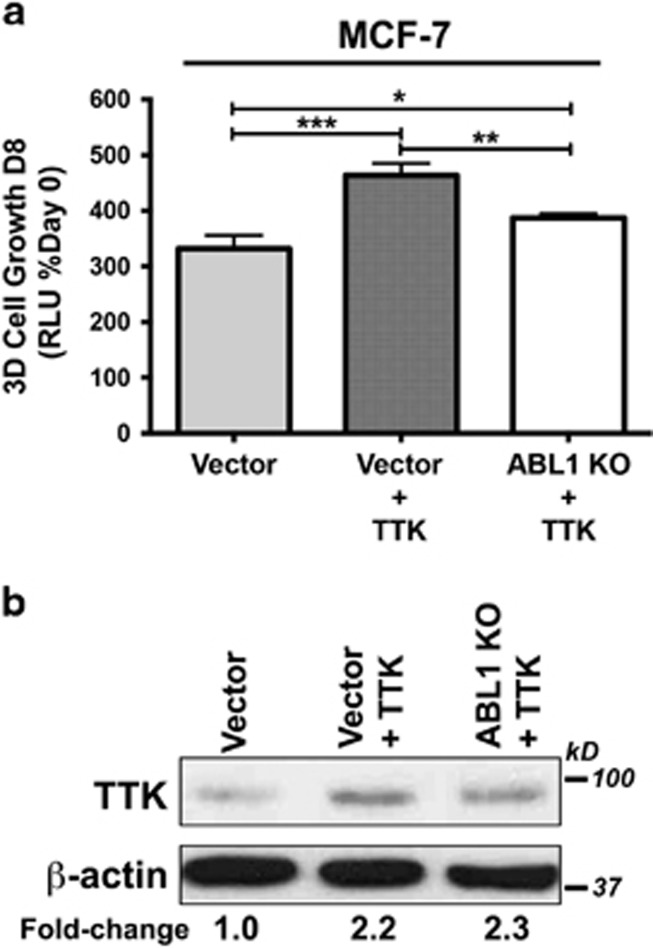
Heterologous TTK expression promotes MCF-7 cell proliferation in part through a c-Abl-dependent mechanism. (**a**) Stable expression of TTK significantly induced the growth of MCF-7 organoids in 3D-cultures, an event that was inactivated in Abl-deficient cells. Longitudinal growth was quantified by firefly bioluminescence. *P<0.05; **P<0.01; ***P<0.001. (**b**) Immunoblot monitoring heterologous TTK expression in MCF-7 cells. Data are the mean (±S.E.) of three independent experiments completed in triplicate

TTK is a critical regulator of the mitotic spindle assembly checkpoint and insures the faithful segregation of sister chromatids by confirming their correct orientation and attachment to microtubules prior to their separation.^18^ Increased TTK expression has been shown to be protective of aneuploidy in breast cancer, leading to attempts to therapeutically target this protein kinase in TNBCs.^21^ Given our findings that c-Abl and TTK expression are correlated with that of p53, we next hypothesized that heterologous TTK expression in MCF-7 cells would enhance their oncogenic potential by circumventing the tumor suppressive activity of c-Abl, whereas functional disruption of TTK in MDA-MB-231 cells would restore the anti-tumor activities of c-Abl. To test this supposition, we engineered parental and c-Abl-deficient MCF-7 cells to express TTK, as well as inactivated TTK activity in parental MDA-MB-231 cells via either CRISPR/Cas9-mediated gene knockout or administration of small molecule TTK inhibitors. As expected, treating MDA-MB-231 cells with DPH failed to affect their growth in 2D-cultures (Figure 3a), as did singular administration of either the TTK inhibitor, MSP1-IN-3^22^ or the 14-3-3 inhibitor, BV02^23^ (Figure 3a). However, Figures 3b and c show that combining TTK inactivation with DPH-mediated c-Abl activation significantly decreased the growth of MDA-MB-231 cells in 2D- and 3D-cultures. Likewise, CRISPR/Cas9-mediated TTK knockout significantly reduced MDA-MB-231 cell growth in 2D- and 3D-cultures, as well as sensitized them to DPH-mediated c-Abl activation (Figures 4a–c). Interestingly, Figure 4d shows that functional disruption of TTK expression had no effect on the subcellular localization of c-Abl, suggesting that c-Abl activation in a TTK-null background stimulates pathways that are distinct from its tumor suppressing functions that arise in the nucleus (Supplementary Figure S2). We also determined that heterologous TTK expression significantly promoted the 3D-proliferation of MCF-7 cells, an event that was significantly attenuated by rendering these cells deficient in c-Abl expression (Figures 5a and b). Collectively, these findings suggest that TTK mediates its oncogenic effects in part through a c-Abl-dependent mechanism that is uncoupled from the activities of 14-3-3 family members.

### Mutant p53 and c-Abl form a cytoplasmic complex in MDA-MB-231 that is dependent upon the kinase activity of c-Abl

**Figure 6:**
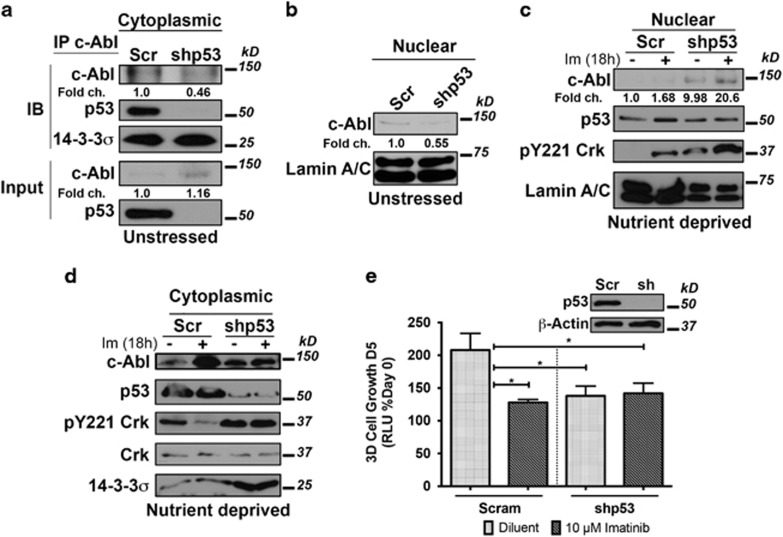
Mutant p53 binds c-Abl in the cytoplasm and is an essential mediator of oncogenic c-Abl signaling MDA-MB-231 cells. (**a**, upper panel) Parent and p53-deficient MDA-MB-231 cells were propagated in complete media (unstressed), at which point c-Abl immunocomplexes captured from the cytoplasmic fractions and probed with antibodies against p53 and 14-3-3*σ*. (*bottom panel)* Differences in starting material were monitored by immunoblotting 5% of the input volume used in capturing c-Abl immunocomplexes. (**b**) Expression of c-Abl and lamin a/c in nuclear fractions of parental and p53-deficient MDA-MB-231 cells propagated in complete media (unstressed). (**c** and **d**) Parental and p53-deficient MDA-MB-231 cells were propagated in serum-free media (nutrient deprived) in the absence or presence of Imatinib (10 *μ*M) for 18 hr as indicated. Afterward, nuclear (**c**) and cytoplasmic (**d**) fractions were prepared and subjected to GST-Crk *in vitro* kinase assay. The extent of Y221-Crk phosphorylation was determined by immunoblotting and used as a surrogate of c-Abl activity. Also shown are the expression levels of c-Abl, p53, the nuclear marker, lamin a/c, and the cytoplasmic marker, *β*-tublin. (**e**) The growth of parental and p53-deficient MDA-MB-231 organoids in the absence or presence of Imatinib (10 *μ*M) was monitored longitudinally by firefly bioluminescence. Inset: immunoblot confirming p53 knockdown. Data are the mean (±S.E.) of three independent experiments completed in triplicate for (**e**) *P<0.05. Images are representative of 3 independent experiments for **a** and **b**, and of three independent experiments for (**c** and **d**)

Molecular profiling and our aforementioned findings clearly show that TNBCs express high levels of mutant p53^24, 25^ (Figure 2); however, the impact of p53 mutations in regulating c-Abl activity remains to be elucidated. It is interesting to note that mutant p53 expressed in TNBCs can form cytoplasmic aggregates that in many respects resemble nuclear prion-like amyloid oligomers and fibrils commonly found in neurodegenerative diseases.^26^ Importantly, these mutant p53 aggregates also sequester wild-type p53 within these tangles, thereby exerting a dominant-negative phenotype over wild-type p53 function.^26^ As such, we hypothesized that mutant p53 aggregates also seed the aggregation of c-Abl within the cytoplasm, thus providing a novel mechanism whereby developing and progressing TNBCs hijack the oncogenic activities of c-Abl. In support of this hypothesis, we found c-Abl and mutant p53 to form cytoplasmic complexes with 14-3-3*σ* (Figure 6a). Interestingly, 14-3-3*σ* remained complexed with c-Abl in the cytoplasm of p53-deficient MDA-MB-231 cells (Figure 6a), suggesting that alternative mechanisms underlie the cytoplasmic retention of c-Abl in TNBCs. Our hypothesis predicts that preventing the formation of cytoplasmic p53:c-Abl complexes should elicit greater nuclear accumulation of c-Abl. As shown in Figure 6b, p53-deficiency failed to impact the subcellular localization of c-Abl in MDA-MB-231 cells propagated in complete media. However, depriving p53-depleted MDA-MB-231 cells of nutrients resulted in the significant nuclear accumulation and activation of c-Abl, an event that was noticeably absent in p53-proficient MDA-MB-231 cells (Figures 6c and d).

Finally, we investigated the impact of Imatinib in altering the subcellular localization of c-Abl and p53 in MDA-MB-231 cells. In doing so, we observed Imatinib administration to elicit robust nuclear accumulation of both c-Abl and p53 expression in parental MDA-MB-231 cells (Figure 6c). Much to our surprise, we also detected nuclear expression of p53 in serum-starved p53-depleted MDA-MB-231 cells (Figure 6c). The fact that shRNA-mediated depletion of p53 is nearly complete in the cytoplasm of these cells (Figure 6d) suggests that measures capable of alleviating mutant p53 expression elicit the release c-Abl from the cytoplasm, thereby enabling this PTK to execute its anti-tumor activities and stabilization of wild-type p53 expression in the nucleus.^27^ In support of this, we asked whether the increase in nuclear c-Abl expression after Imatinib treatment correlated with an increase in its nuclear kinase activity. To do so, we performed a modified *in vitro* kinase assay, which measured the phosphorylation of an artificial c-Abl substrate, GST-Crk^28^ upon the addition of exogenous ATP in cytoplasmic and nuclear extracts from parental and p53-deficient MDA-MB-231 cells. In doing so, we found parental MDA-MB-231 cells to possess lower overall levels of c-Abl activity, which was selectively inhibited by Imatinib in the cytoplasmic, but not in the nucleus (Figures 6c and d). Interestingly, administering Imatinib to p53-deficient MDA-MB-231 cells was ineffective at inhibiting c-Abl activity in either subcellular locale (Figures 6c and d) or at attenuating MDA-MB-231 cell 3D-proliferation (Figure 6e). Collectively, these findings suggest that mutant p53 may engender the cytoplasmic localization and oncogenic activities of c-Abl during the development and progression of distinct TNBCs.

## Discussion

Our previous findings identified c-Abl as a robust and multifaceted tumor suppressor whose anticancer activities are dependent upon the mutational and expression status of p53.^10, 11^ The mitotic kinase, TTK, is overexpressed in mutant p53-expressing TNBCs and has been shown to regulate the cellular localization of c-Abl in response to oxidative stress.^14, 15, 16, 17, 18, 21^ Herein, we also established p53 expression (wild-type or mutant) as an essential determinant for the correlation between c-Abl and TTK expression across all breast cancer subtypes. Importantly, this correlation was most relevant in basal-like breast cancers, whose loss of TTK expression inhibited their proliferation and sensitized them to DPH-mediated c-Abl activation. In contrast, engineering luminal A cells to overexpress TTK induced their growth in a mechanism that was in part dependent upon the expression of c-Abl. These results are particularly interesting with respect to the importance of p53 expression in regulating that of TTK and c-Abl, as this correlation predicts that depletion of TTK expression would correlate with an increase in c-Abl expression and *vice versa*. Accordingly, we recently determined that inhibiting TTK activity does indeed promote the stabilization and accumulation of c-Abl expression (CDM and WPS, unpublished results). Ultimately, loss of TTK expression failed to impact the subcellular localization of c-Abl in TNBCs, suggesting that TTK-deficiency may serve in coupling c-Abl to novel regulators of cell-fate, which are separate from its known anticancer activities that transpire in the nucleus. Accordingly, cytoplasmic c-Abl has been shown to maintain proper balance of Crk/p130CAS complexes that traditionally form after integrin and growth factor receptor engagement operant in stimulating cell migration and survival.^28^ Cytoplasmic c-Abl participates in these events by functioning in a negative feedback loop coupled to the phosphorylation of Crk at Y221-Crk, thereby limiting cell migration and survival.^28^ Future studies need to determine whether TTK inactivation selectively engages the apoptosis-promoting activities of c-Abl in normal and malignant MECs.

Although TTK initially represented a logical culprit responsible for regulating the cellular localization and function of c-Abl in mutant p53-positive TNBCs, our findings eventually excluded TTK as a direct mediator of these events. Given the recently established prion theory related to mutant and wild-type p53 aggregation,^29^ we asked whether mutant p53 aggregates also inhibit the tumor suppressing functions of c-Abl. Accordingly, we demonstrated the formation of cytoplasmic complexes that contained mutant p53, c-Abl, and 14-3-3*σ*, which presumably contributes to the ability of mutant p53 expression in governing the sensitivity of MDA-MB-231 cells to Imatinib and its inhibition of c-Abl activity. The physical interaction between c-Abl and p53 is known to regulate DNA damage responses, whereas that between 14-3-3*σ* and p53 is necessary to stabilize and oligomerize p53 during anti-proliferative programs.^30^ Interestingly, we observed 14-3-3*σ* to remain constitutively associated with c-Abl in a manner independent of mutant p53 status, suggesting that (i) the cytoplasmic retention of c-Abl in MDA-MB-231 cells occurred through unconventional mechanisms, and (ii) 14-3-3*σ* and c-Abl may serve in coordinating the regulation of p53 during DNA damage responses.

It should be noted that the binding of 14-3-3*σ* to ‘Abl’ primarily relates to BCR-ABL1, and as such, evidence supporting a role for 14-3-3*σ* in mediating the cytoplasmic localization of ‘Abl’ remains tenuous. Indeed, a recent study demonstrated that the retention of BCR-ABL1 in the cytoplasm is independent of 14-3-3*σ*, and instead transpires through a bimodal mechanism involving its (i) F-actin-binding domain, and (ii) an autophosphorylation-dependent conformational change that masks the nuclear localization sequences (NLSs) in ABL1.^31^ Importantly, Imatinib administration abrogates the autophosphorylation activity of ABL1, thereby relieving the inhibitory conformation within ABL1 and exposing its NLSs. Along these lines, Imatinib resistance can emerge in response to PTK domain mutations that prevent Imatinib-mediated unmasking of NLS sequences, thus preventing nuclear entry of BCR-ABL1 in response to Imatinib.^31^ Our findings suggest the presence of an analogous mechanism in some TNBCs, wherein mutant p53 binds c-Abl and masks its NLSs. Moreover, we observed nutrient deprivation to be sufficient in eliciting robust nuclear accumulation of c-Abl in p53-depleted MDA-MB-231 cells, as well as the ability of Imatinib to be capable of driving c-Abl and p53 into the nucleus of parental MDA-MB-231, indicating that mutant p53 does indeed sequester c-Abl by occluding its NLSs. Even more remarkably, we observed markedly elevated c-Abl activity upon its arrival to the nucleus in MDA-MB-231 cells, even in Imatinib-treated MDA-MB-231 cells undergoing proliferative arrest. Finally, although expression of shRNA against p53 effectively depleted its expression in the cytoplasm, we nonetheless readily detected p53 expression in the nuclei of nutrient-deprived cells, suggesting that the formation of c-Abl:p53 complexes protects and stabilizes a pool of nuclear p53.^32^

In summary, our findings are the first to define mutant p53 as a direct regulator of oncogenic c-Abl signaling in MDA-MB-231 cells. It should be noted that the binding of c-Abl to the C terminus of wild-type p53 is well established, and necessary for proper transactivation and growth suppression by p53.^33, 34^ Similarly, deletion of the p53-binding domain of c-Abl prevents the transactivation of p53 and its ability to inhibit proliferative programs.^33^ Collectively, it stands to reason that dysregulation of either c-Abl or p53 disrupts their respective tumor suppressive functions, leading to the development and progression of TNBCs (e.g., MDA-MB-231 cells). Future studies need to extend these analyses to other breast cancer models, and to investigative the efficacy of combining standard-of-care chemotherapies with c-Abl-targeted therapies as a specific strategy to eliminate TNBCs that house mutant p53.

## Materials and methods

### Cell lines and reagents

Luminal MCF-7 and triple-negative MDA-MB-231 cells were obtained from ATCC and cultured as previously described.^35^ MDA-MB-231 cells engineered to express firefly luciferase were previously described,^35^ whereas luciferase-expressing MCF-7 cells were generated by their transduction with lentiviral particles that encoded for firefly luciferase and subsequent selection in blasticidin (5 *μ*g/ml). Stable expression of TTK in MCF-7 cells was accomplished by transient transfection of the human TTK cDNA (2.5 *μ*g; NM_003318, Product #RC200093; Origene, Rockville, MD, USA), at which point stable polyclonal populations were isolated by neomycin (200 mg/ml; Calbiochem, Billerica, MA, USA) selection over a span of 14 days. GlaxoSmithKline (GSK) kindly provided the small molecule allosteric c-Abl activator DPH.^12^

### Functional disruption of target gene expression

In some experiments, target gene expression was disrupted by Clustered Interspaced Short Palindromic Repeat/Cas9 (CRISPR/Cas9)-mediated gene knockout. Accordingly, guide RNA (gRNA) oligonucleotides directed towards ABL1 and TTK genes were cloned into the lentiCRISPRv2 plasmid as described.^36, 37^ ABL1 and TTK gRNA sequences were determined utilizing http://crispr.mit.edu and https://chopchop.rc.fas.harvard.edu/, respectively. gRNA sequences are listed in Supplementary Table S1. MCF-7 and MDA-MB-231 cells were transduced with the indicated CRISPR/Cas9 ABL1 or TTK lentiviral particles and were selected over a span of 14 days in puromycin (5 *μ*g/ml). Gene knockout was determined by immunoblotting of the encoded protein. Stable expression of CST-Abl in ABL1 knockout MCF-7 and MDA-MB-231 cells was accomplished through transient transfection of the ecotropic receptor prior to transduction with CST-Abl retroviral particles as described previously.^10^

In some experiments, target gene expression was disrupted by shRNA-mediated knockdown. Accordingly, human p53 expression was functionally disrupted by transducing MDA-MB-231 cells with lentiviral particles that encoded for shRNA against p53 (pLV-shp53-bleo^38^) as described previously.^10^ The pLV-shp53-bleo construct was kindly provided by Dr. Mark Jackson.

### Invasion assay

The ability of MCF-7 and MDA-MB-231 cells (50 000 cells/well) to invade reconstituted basement membranes was measured in modified Boyden chambers by utilizing 2% serum as the chemoattractant in the absence or presence of DPH (40 *μ*M) as described previously.^39^

### Immunoblotting

Whole-cell lysates (WCEs) were prepared as previously described.^40^ Briefly, MCF-7 and MDA-MB-231 cells were seeded into 6-well plates (500 000 cells per well) and allowed to adhere overnight, at which point they were treated for the indicated times in the presence or absence of the various activators or inhibitors listed in Supplementary Table S2. Detergent-solubilized WCEs were prepared by lysing the cells in RIPA buffer (50 mM Tris, 150 mM NaCl, 6 mM sodium deoxycholate, 1% NP-40, 0.1% SDS, pH 7.4) supplemented with protease inhibitor cocktail (Sigma, St. Louis, MO, USA) and phosphatase inhibitors (10 mM sodium orthovanadate, 40 mM *β*-glycerophosphate, 20 mM NaF), and subsequently were clarified by microcentrifugation. Afterward, 30 *μ*g of WCE were fractionated through 10% SDS-PAGE gels, transferred electrophoretically to PVDF-P-membranes, and immunoblotted with the antibodies described in Supplementary Table S2.

### Cell fractionation and immunoprecipitation

MDA-MB-231 and MCF-7 cells were cultured on 10 cm plates and harvested upon reaching 70% confluency in Buffer A (10 mM HEPES, 10 mM KCL, 0.1 mM EDTA and 0.004% Nonidet P-40, pH 7.9) supplemented with a protease inhibitor cocktail (Sigma). Afterward, the WCE was clarified by microcentrifugation yielding a cytoplasmic fraction. The remaining pellet was washed extensively with PBS and then lysed in Buffer B (20 mM HEPES, 400 mM NaCl, 1 mM EDTA, and 10% glycerol, pH 7.9) supplemented with protease inhibitor cocktail and vortexed at maximum speed for 2 h at 4 °C. Afterward, the nuclear pellet was clarified by microcentrifugation. Cytoplasmic and nuclear fractions were analyzed by immunoblotting with anti-c-Abl, anti-p53, and anti-14-3-3α antibodies (Supplementary Table S2). In addition, cytoplasmic and nuclear fractions were immunoprecipitated as described^41^ with antibodies against 14-3-3*σ*, p53, and c-Abl, and subsequently were subjected to immunoblot analysis of c-Abl, p53, and 14-3-3*σ* as indicated (Supplementary Table S2). Anti-lamin A/C and anti-*β*-tubulin antibodies were used as loading controls for nuclear and cytoplasmic fractions, respectively (Supplementary Table S2).

### *In vitro* kinase assay

MDA-MB-231 cells were incubated in the absence or presence of 10 *μ*M of Imatinib in serum-free media as indicated. Afterward, the resulting nuclear and cytoplasmic fractions were isolated, at which point 15 *μ*g of each fraction was incubated in kinase assay buffer^10^ supplemented with 2 *μ*g of the artificial c-Abl substrate, GST-Crk (Product # 14-468, EMD Millipore, Billerica, MA, USA). The protein kinase reactions were initiated by the addition of ATP (5 *μ*M) and allowed to proceed under continuous rotation for 60 min at 37 °C. The reactions were terminated by addition of 4 × sample buffer^42^ and subsequently prepared for anti-phospho-Y221-Crk (1:500; Abcam, Cambridge, MA, USA) immunoblotting to monitor c-Abl kinase activity. Total anti-Crk (1:1000; Abcam, Cambridge, MA, USA) antibody was used as a loading control.

### 2D-proliferation and 3D-organotypic culture outgrowth assays

MDA-MB-231 cells (1000 cells per well) were cultured in 96-well plates in the presence of BV02 (1.0 *μ*M; Sigma-Aldrich, Product # 01040), MPS1-IN-3 (0.5 *μ*M; Sigma-Aldrich, Product # SML0898) or DPH (40 *μ*M) and their 2D-proliferation was analyzed utilizing the phase-contrast analysis mode on an IncuCyte ZOOM (Essen Bio-Science; Ann Arbor, MI, USA). Briefly, phase-contrast images were taken at the start of treatment and at 60 h, at which their final phase-contrast readings were normalized to initial phase confluence and statistical comparisons between the indicated treatments were made.

The ‘on-top’ method of 3D-organotypic culturing was utilized as described.^35, 43^ Briefly, MCF-7 and MDA-MB-231 cells (1000 cells per well) were cultured in 96-well plates on 50 *μ*l Cultrex cushions (Trevigen, Gaithersburg, MD, USA) in growth media supplemented with 5% Cultrex. Where indicated, the cells were treated with MPS1-IN-3 (0.5 *μ*M), DPH (20 *μ*M), or Imatinib mesylate (10 *μ*M; LC Laboratories, Woburn, MA, USA), The media/Cultrex solution was replaced on days in which bioluminescence was being quantified. Biolouminescence was measured longitudinally after the addition of d-luciferin (Gold Biotechnology, St. Louis, MO, USA) as described.^35, 40, 43^

### Statistical analyses

Data are the mean (±S.E.) obtained from at least three independent experiments. Statistical values were defined using an unpaired Student’s *t*-test with a *P*-value of <0.05 considered significant.
