# Supplementary material for: Mutant p53 dictates the oncogenic activity of c-Abl in triple-negative breast cancers
Source: Cell Death Dis. 2017 Jun 29;8(6):e2899–. doi: 10.1038/cddis.2017.294 (PMC5520943; doi:10.1038/cddis.2017.294)
Supplement: Supplementary Materials and Data [file cddis2017294x1.pdf]

Supplementary Materials and Data

Mutant p53 Dictates the Oncogenic Activity of c-Abl in Triple-Negative Breast Cancers

Chevaun D. Morrison[1], Jenny C. Chang[2], Ruth A. Keri[3] and William P. Schiemann[1]

Author Affiliations:
Case Comprehensive Cancer Center, Case Western Reserve University, Cleveland, OH 44106. [2]Houston Methodist Research Center, Houston, TX 77030. [3]Department of Pharmacology, Case Western Reserve University, Cleveland, OH 44106.

Address Correspondence to:
William P. Schiemann, Case Comprehensive Cancer Center, Case Western Reserve University, Wolstein Research Building, Room 2131, 2103 Cornell Road Cleveland, OH 44106 Phone: 216-368-5763. Fax: 216-368-1166. E-mail: william.schiemann@case.edu

Running Title: Mutant p53 Dictates c-Abl Function in TNBCs

Key Words: Breast cancer; c-Abl; p53; Signal transduction; triple-negative breast cancer; TTK.

Conflict of Interest: The authors declare no conflict of interest.

Supplementary Materials and Methods

Supplementary Table S1: CRISPR/Cas9 Guide Oligonucleotide Sequences

Target	Application	Sequence (5' to 3')
ABL1	Forward sgRNA	5'- CACCGAGAGATCAAACACCCTAACC
ABL1	Reverse sgRNA	5'- AAACGTACGGGGAGGTGTACGAGGC
TTK	Forward sgRNA	5'- CACCGAAGTAGTCACGTGCATCATC
TTK	Reverse sgRNA	5'- AAACGATGATGCACGTGACTACTTC

Supplementary Table S2: Immunoblotting Antibodies

Antibody	Dilution	Supplier
c-Abl	1:1000 WB; 1:200 IP	Cell Signaling Technologies (#2682; Danvers, MA, USA)
Mps1/TTK	1:1000	Abcam (#Ab11108; Cambridge, United Kingdom)
p53	1:1000 WB; 1:200 IP	Cell Signaling Technologies (#2524)
14-3-3σ	1:1000 WB; 1:200 IP	Abcam (#Ab1423)
Lamin A/C	1:2000	Santa Cruz Biotechnologies (#SC-7293; Santa Cruz, CA, USA)
Phospho-Y221-Crk	1:500	Abcam (#Ab51222)
Total Crk	1:1000	Abcam (#Ab45136)
β-actin	1:10,000	Sigma-Aldrich (#A5441; St. Louis, MO, USA)
β-tubulin	1:2000	Sigma-Aldrich (#T4026)

Shown are the antibodies and dilutions used to visualize the indicated proteins. Also provided are the product #'s and vendors where these reagents were obtained.

Supplementary Figure 1: Morrison *et al*

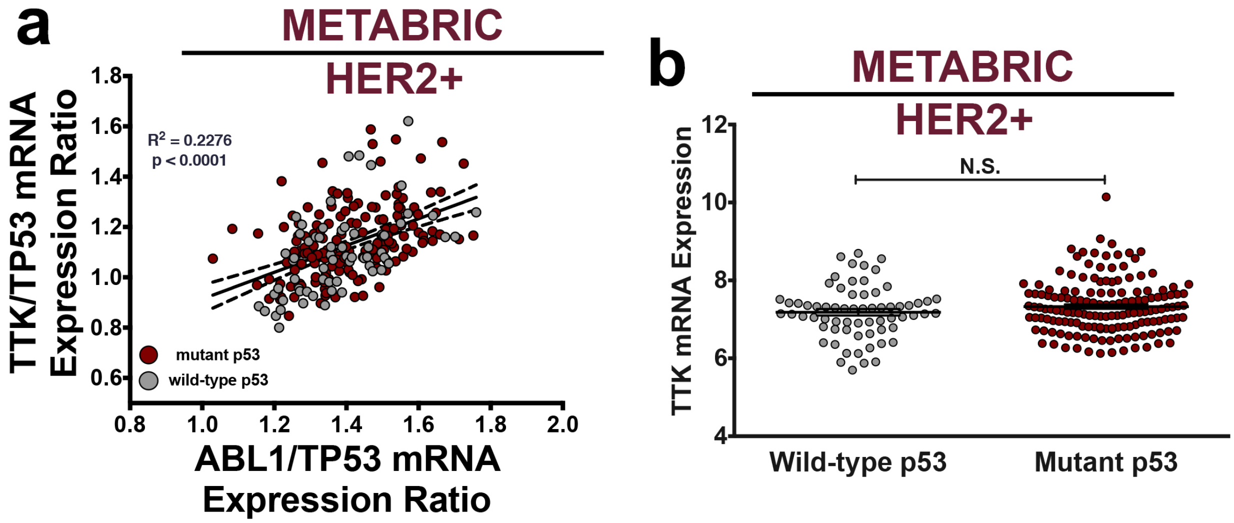

Supplementary Figure S1 p53 expression is a critical determinant of TTK and c-Abl expression in HER2$^+$ breast cancers. (**a**) Comparison of TTK expression in wild-type and mutant p53-positive HER2$^+$ BC patients from the METABRIC BC data set. (**b**) Linear regression analysis of the HER2$^+$ subtype of the METABRIC BC data set comparing the correlation between TTK:TP53 and ABL1:TP53 expression ratios.

Supplementary Figure S2: Morrison *et al*

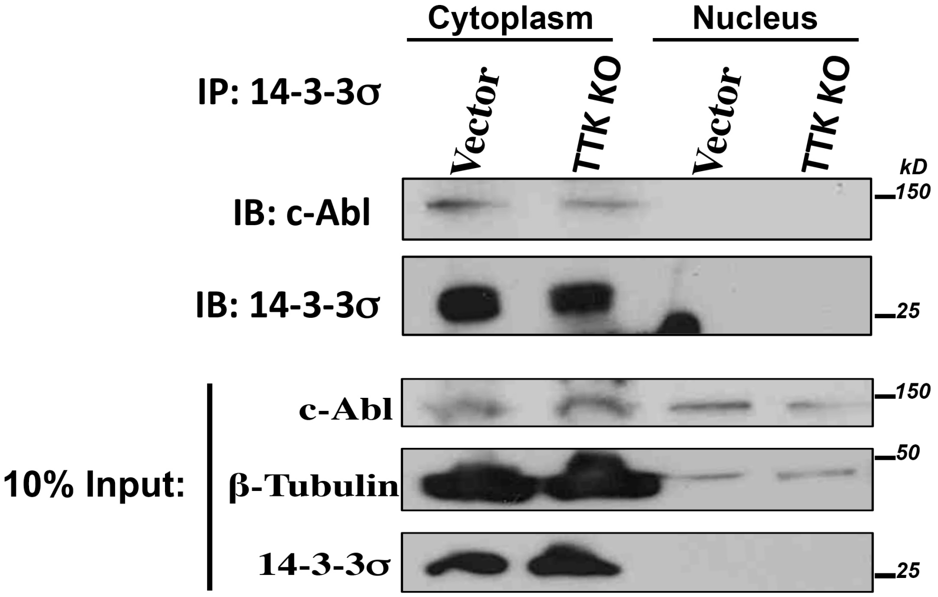

Supplementary Figure S2 CRISPR/Cas9-mediated TTK knockout fails to impact the cellular localization of c-Abl in MDA-MB-231 cells. (**Top**) 14-3-3α immuneprecipitates (IP) were captured from cytoplasmic and nuclear fractions of parental and TTK-deficient MDA-MB-231 cells, and subsequently immunoblotted for c-Abl and 14-3-3σ as indicated. (**Bottom**) Differences in protein loading were monitoring by immunoblotting 10% aliquots of the protein input with antibodies against c-Abl, β-tubulin, and 14-3-3σ as shown. Data are representative images from 2 independent experiments.
